# Bacterial Tradeoffs in Growth Rate and Extracellular Enzymes

**DOI:** 10.3389/fmicb.2019.02956

**Published:** 2019-12-20

**Authors:** Kelly I. Ramin, Steven D. Allison

**Affiliations:** ^1^Department of Ecology and Evolutionary Biology, University of California, Irvine, Irvine, CA, United States; ^2^Department of Earth System Science, University of California, Irvine, Irvine, CA, United States

**Keywords:** extracellular enzyme, leaf litter, life history strategy, maximum growth rate, phylogenetic conservation, soil bacteria, trait tradeoff

## Abstract

Like larger organisms, bacteria possess traits, or phenotypic characteristics, that influence growth and impact ecosystem processes. Still, it remains unclear how these traits are organized across bacterial lineages. Using 49 bacterial strains isolated from leaf litter in Southern California, we tested the hypothesis that bacterial growth rates trade off against extracellular enzyme investment. We also tested for phylogenetic conservation of these traits under high and low resource conditions represented, respectively, by Luria broth (LB) and a monomer-dominated medium extracted from plant litter. In support of our hypotheses, we found a negative correlation between the maximum growth rate and the total activity of carbon-, nitrogen-, and phosphorus-degrading extracellular enzymes. However, this tradeoff was only observed under high resource conditions. We also found significant phylogenetic signal in maximum growth rate and extracellular enzyme investment under high and low resource conditions. Driven by our bacterial trait data, we proposed three potential life history strategies. *Resource acquisition* strategists invest heavily in extracellular enzyme production. *Growth* strategists invest in high growth rates. Bacteria in a third category showed lower potential for enzyme production and growth, so we tentatively classified them as *maintenance* strategists that may perform better under conditions we did not measure. These strategies were related to bacterial phylogeny, with most growth strategists belonging to the phylum Proteobacteria and most maintenance and resource acquisition strategists belonging to the phylum Actinobacteria. By accounting for extracellular enzyme investment, our proposed life history strategies complement existing frameworks, such as the copiotroph-oligotroph continuum and Grime’s competitor-stress tolerator-ruderal triangle. Our results have biogeochemical implications because allocation to extracellular enzymes versus growth or stress tolerance can determine the fate and form of organic matter cycling through surface soil.

## Introduction

Life history strategies are suites of traits, or phenotypic characteristics, that have evolved to optimize fitness under specific environmental conditions. Multiple life history classifications have been proposed, beginning with r- and K-strategists for plants and animals (Pianka, 1970). Later, microbes were suggested to fall along a life history continuum from copiotrophs to oligotrophs (Koch, 2001; Fierer et al., 2007). Copiotrophs are generalists with high maximum growth rate (*μ*_max_), relatively large cell and genome sizes, and adaptations for rapid growth in nutrient-rich environments, such as high rRNA gene copy number and high nutrient uptake capacity. Oligotrophs, on the other hand, are specialists with a low *μ*_max_, smaller cell and genome sizes, and adaptations such as high substrate affinity to support growth in nutrient-poor environments (Lauro et al., 2009).

More recently, microbial ecologists have also begun to apply Grime’s triangle of plant competitor-stress tolerator-ruderal (CSR) strategies to microbial systems (Grime, 1977; Krause et al., 2014; Fierer, 2017). Microbial stress tolerators are thought to have traits similar to oligotrophs in terms of growth rate and substrate affinity. Competitors should have low tolerance of stress and disturbance as well as traits that enhance competitive ability, such as antibiotic and siderophore production. Ruderals should take advantage of disturbed niches with rapid growth and spore formation. While this emerging framework is promising, it remains unclear what are the key strategies and underlying traits for microbes. For instance, existing strategy concepts do not consider extracellular enzyme production, a crucial trait for microbial resource acquisition with implications for carbon and nutrient cycling in ecosystems (Allison et al., 2011).

Extracellular enzymes are produced by microbes to degrade complex organic matter into useable products that can be taken up across the cell membrane. These enzymes are required to access resources in chemically complex polymers, but they are also costly to synthesize and secrete (Koch, 1985). As a result, cellular investment in enzyme production may trade off against other metabolic processes (Carlson and Taffs, 2010). Enzyme production traits—and how they trade off with other resource allocation traits—are therefore potentially important components of microbial life history strategies. Furthermore, the resource allocation traits of microbial life histories can play a pivotal role in ecosystem processes (Schimel and Schaeffer, 2012). For example, increasing enzyme production may lower carbon use efficiency, resulting in higher rates of respiration and decreased soil carbon storage (Six et al., 2006).

There is growing interest in using traits to link microbial community composition with ecosystem functioning (Martiny et al., 2015). For these efforts, it would be convenient if microbial life history strategies were phylogenetically conserved because broad suites of ecologically relevant traits could then be determined based on phylogeny. Traits involving few genes, such as the production of specific extracellular enzymes, tend to vary toward the tips of the phylogenetic tree, whereas complex traits involving many genes are conserved in deeper clades (Martiny et al., 2013, 2015; Zimmerman et al., 2013). Yet, it remains uncertain whether microbial life history strategies exhibit shallow or deep phylogenetic conservation.

With this study, we aimed to address (1) whether tradeoffs exist among bacterial growth and enzyme production; (2) how these potential tradeoffs fit into an overall strategy; (3) how the tradeoffs are influenced by resource availability; and (4) if these patterns are phylogenetically conserved. Across broad taxonomic groups, we hypothesized that bacterial extracellular enzyme investment trades off against maximum growth rate. Further, we hypothesized that the tradeoff should weaken under high resource conditions because bacteria will have more resources to invest in multiple cellular processes. Finally, we hypothesized that growth and extracellular enzyme traits should be phylogenetically conserved due to evolutionary constraints on resource allocation. To test these hypotheses, we analyzed extracellular enzyme activity, extracellular protein production, and growth rate on two different substrate types in bacterial strains isolated from plant litter.

## Materials and Methods

### Bacterial Strains

We analyzed 49 bacterial strains previously isolated from plant litter in a Mediterranean grassland ecosystem in Southern California (Potts et al., 2012; Mouginot et al., 2014; Matulich and Martiny, 2015). Isolation media included Luria broth (LB), plant litter broth (PB; see details below), or dilute nutrient broth (DNB; Supplementary Material). The isolated strains include 18 representatives from the Phylum Proteobacteria, 27 from Actinobacteria, and 4 from Bacteroidetes.

### Growth and Storage Conditions

All strains were initially stored at −80°C in glycerol stocks. These stocks were used to streak onto LB agar plates. Once colonies formed, one colony from each strain was chosen to inoculate a corresponding flask of liquid LB, which, upon reaching exponential growth, was then used to inoculate further flasks to produce growth curves and supernatant for enzyme and protein assays. All flasks were incubated at 28°C on a shaking platform. LB was chosen because these bacteria were shown previously to grow in this medium, and it represents nutrient-rich conditions, likely supporting the maximum growth rate possible for many of these strains.

We also cultured strains on PB that was made using grass litter from the site where the strains were originally isolated. Litter was dried, ground, and added to deionized water. After 6 h of heated stirring, the litter was removed using centrifugation, and the medium was filtered and sterilized by autoclaving. All PB was made using the same litter batch to ensure consistent nutrient conditions. Although we did not analyze PB directly, metabolomic analysis on cold water extracts from grass litter suggests that PB composition is dominated by amino acids, organic acids, nucleosides, and other low molecular weight compounds (Malik et al., 2019b). Therefore, compared to LB, PB is likely more representative of the environment from which the bacteria were originally isolated. In preliminary growth assays, PB was found to support growth rates typically associated with nutrient poor conditions; that is, most strains had an instantaneous specific growth rate (*μ*) less than 5% of maximum potential growth rate as measured on LB (Konopka, 2000).

Growth curves were performed in triplicate, with time intervals between measurements dependent upon rate of growth for the individual strain. For enzyme and protein assays, each strain was grown in quintuplicate for 14 h, just before the fastest growing strains reached stationary phase. At this time, the optical density (OD₆₀₀) was measured, the cultures were centrifuged, and the supernatant was collected and frozen at −20°C until further processing. For these analyses, it was important to measure enzyme activity and protein production during active growth to determine the overall investment relative to growth.

### Growth Rate Determination

Spectrophotometer measurements (OD₆₀₀) were made using 96-well plates and a BioTek Synergy 4 plate reader. Due to variation in growth curve shape across strains, we used nonparametric smoothing splines in the “growthrates” R package (Kahm et al., 2010) to determine the maximum growth rate μ for each strain in both broths (Supplementary Figure 1). For strains that displayed a biphasic growth pattern, μ was calculated with the portion of the curve corresponding to the initial growth phase.

### Enzyme and Protein Assays

Hydrolytic enzymes were assayed using the methods by German et al. (2011) with 50 mM maleate buffer, pH 6.0. We analyzed C-targeting enzymes α-glucosidase, β-glucosidase, cellobiohydrolase, and β-xylosidase; P-targeting acid phosphatase; and N-targeting leucine aminopeptidase and N-acetyl-glucosaminidase. Enzyme activities were calculated based on the mean linear change in fluorescence readings taken every 30 min for 2 h. Fluorescence readings were converted to nmol based on 4-methylumbelliferone or 7-amino-4-methylcoumarin standards. Enzyme activities were expressed as nmol product released h^−1^ mg^−1^ biomass assuming that an optical density of 1.0 corresponds to 0.39 mg ml^−1^ bacterial biomass (Glazyrina et al., 2010).

Lowry protein assays were carried out to complement the extracellular enzyme data as an additional metric of extracellular investment. Protein was expressed as μg protein mg^−1^ biomass.

### Phylogeny

Partial sequences of the 16S rRNA gene were obtained for each strain with the Sanger technique as described previously (Mouginot et al., 2014). Sequences were approximately 700 base pairs in length and included the V2 through V4 regions. We used SILVA to verify taxonomic identification against the EMBL, LTP, RDP, SILVA, and GenBank databases; however, the only complete set of taxonomic identification was retrieved from GenBank, with the sequence identity from GenBank being supported by the other databases. We aligned our sequences with the online MAFFT algorithm based on fast Fourier transform^1^ (Katoh and Standley, 2013). After minor manual curation, we used the PHYLIP v. 3.695 software package (Felsenstein, 2005) to create a majority-extended consensus phylogenetic tree from the alignment with default maximum likelihood parameters. The tree and associated trait data were visualized with iTOL v.4.4.2^2^ (Letunic and Bork, 2019). Sequences were deposited in GenBank under accession numbers MN309648–MN309696.

### Statistical Analysis

We assigned strains to trait-based strategies based on growth rate and the sum of all biomass-specific enzyme activities for each strain in LB. Strains with *μ* > 0.389 were assigned to the *growth* strategy; strains with total enzyme activity >9.16 nmol h^−1^ mg^−1^ biomass were assigned to the *resource acquisition* strategy; all remaining strains were assigned to the *maintenance* strategy. These cutoffs are arbitrary but were chosen based on the data to reflect the lack of strains with high-growth, high-enzyme strategies.

Analysis of the phylogenetic signal of traits based on Blomberg’s *K* and Pagel’s *λ* was done using the “phylosignal” package v.1.2 in R (Keck et al., 2016). To perform these calculations, a nominal amount of branch length (1/10 the length of the smallest non-zero terminal branch) was added to terminal branches with zero length. Zero branch lengths arose from analyzing some strains that were nearly indistinguishable from one another based on 16S sequences. Because we could not directly test the phylogenetic conservation of strategy, we used a Pearson’s *χ*^2^ test to determine whether our strategy categorization was correlated with phylum or class.

Because most trait data were non-normally distributed, we used the Wilcoxon Rank Sum Test to test for differences in trait values between LB and PB. Spearman rank correlations were performed to test for relationships between traits. We used the “ape” package v.5.2 in R to verify significant trait correlations in the presence of phylogenetic signal. Enzyme and protein values were (log+1)-transformed prior to phylogenetic analyses.

## Results

### Growth Rates

**Figure 1:**
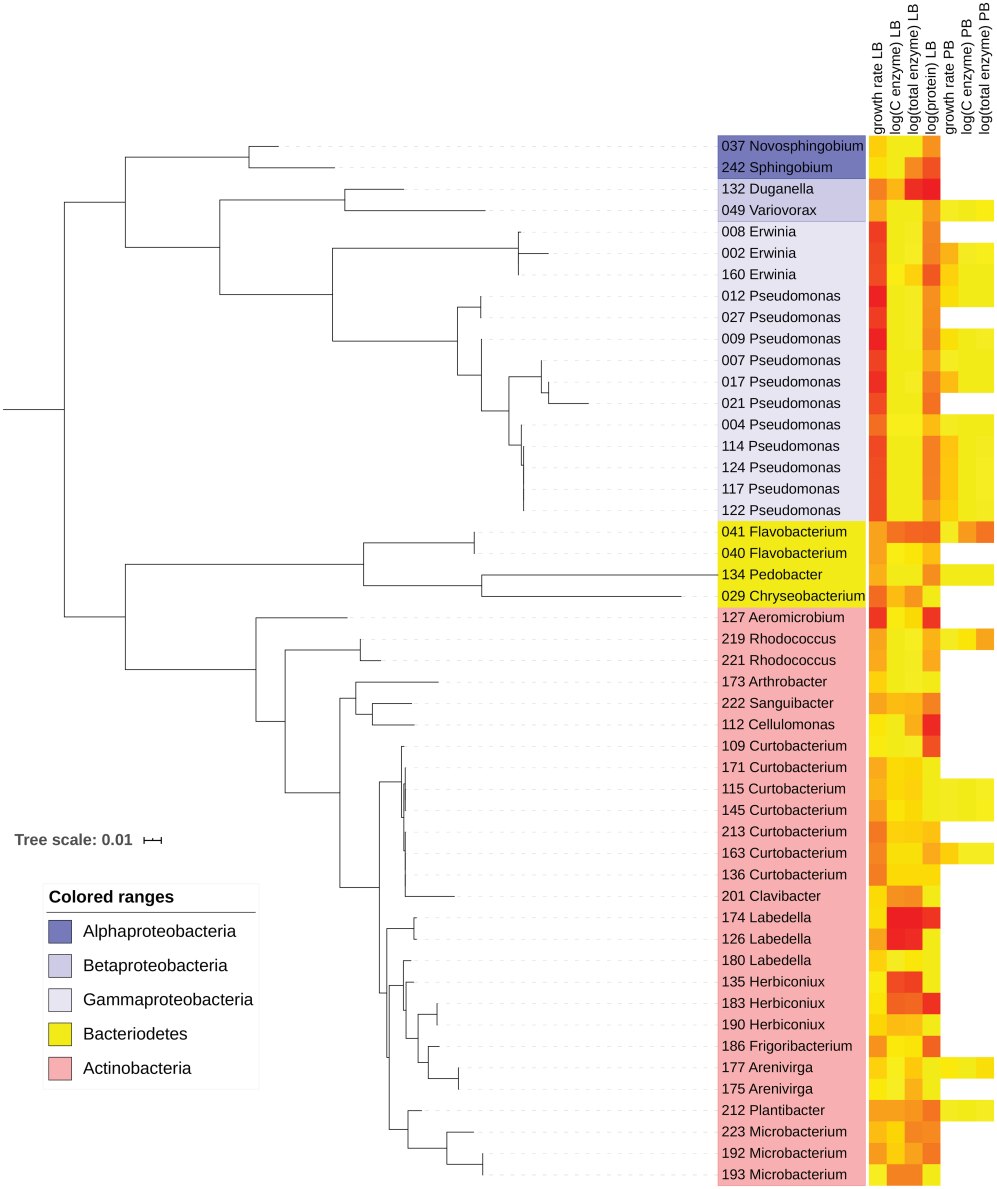
Maximum-likelihood phylogenetic tree with 49 bacterial strains included in the study. Leaves show strain number and likely genus based on GenBank matches. Heatmap indicates relative magnitudes of growth rate, enzyme, and protein traits in Luria broth (LB) and plant litter broth (PB). Yellow corresponds to the minimum value, and red corresponds to the maximum value within each trait.

**Figure 2:**
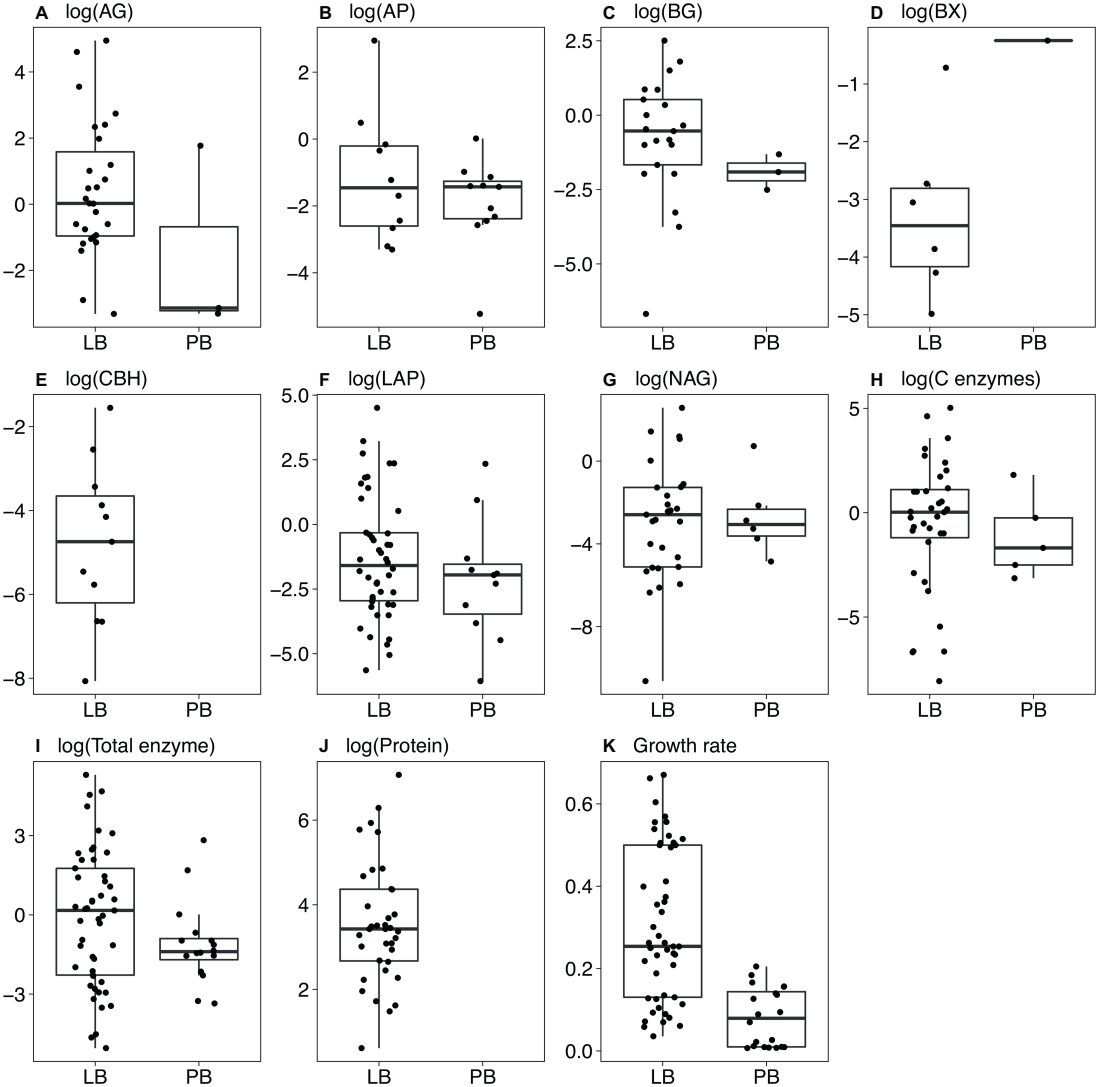
Boxplots of trait values for bacterial strains growing on Luria broth (LB) and plant litter broth (PB). Only non-zero values are shown. **(A)** α-glucosidase; **(B)** acid phosphatase; **(C)** β-glucosidase; **(D)** β-xylosidase; **(E)** cellobiohydrolase; **(F)** leucine aminopeptidase; **(G)** N-acetyl-glucosaminidase; **(H)** the sum of AG, BG, BX, and CBH; **(I)** the sum of all enzyme activities; **(J)** protein level in the culture supernatant; **(K)** maximum growth rate (h^−1^). Boxes indicate the median and first and third quartiles. Whiskers indicate the data range, not including outlying points. **(A)** through **(I)** show (log+1)-transformed units of nmol mg^−1^ biomass h^−1^, and **(J)** shows (log+1)-transformed units of μg protein mg^−1^ biomass.

On LB, maximum growth rates averaged 0.201 h^−1^ for Actinobacteria, 0.284 h^−1^ for Bacteroidetes, and 0.464 h^−1^ for Proteobacteria. Most of the Proteobacteria that grew on LB belonged to the class Gammaproteobacteria (Figure 1; Supplementary Material). Of the 49 strains that grew on LB, only 20 grew on PB, including 6 Actinobacteria, 12 Proteobacteria, and 2 Bacteroidetes. Maximum growth rates were significantly higher on LB than PB for each individual strain and overall (*p* < 0.001, Wilcoxon test), ranging from 0.036 to 0.671 h^−1^ on LB and 0.007 to 0.205 h^−1^ on PB (Figure 2K).

### Extracellular Enzyme Activity and Protein

Total enzyme activity for strains growing on LB ranged from 0.0064 nmol mg^−1^ biomass h^−1^ for Alphaproteobacteria strain 37 to 201 nmol mg^−1^ biomass h^−1^ for Actinobacteria strain 174 (Figure 2I). Total activities were lower on PB (*p* = 0.012, Wilcoxon test), ranging from 0 (four strains) to 16.9 nmol mg^−1^ biomass h^−1^ for Flavobacteria strain 41. However, some individual strains had greater total enzyme activity on PB relative to LB. For example, Actinobacteria strain 219 increased its activity from 0.32 nmol mg^−1^ biomass h^−1^ on LB to 5.42 nmol mg^−1^ biomass h^−1^ on PB (Supplementary Material).

Most strains did not produce all enzyme classes (Figure 2). Only five strains growing on PB had detectable C-degrading enzyme activity (Figure 2H). Eleven strains produced P-acquiring AP on PB versus 10 on LB (Figure 2B). When including strains with zero activity, average AP activity was greater on PB (*p* = 0.008, Wilcoxon test), suggesting a higher demand for phosphorus on PB. Although average N-acquiring enzyme activities (LAP and NAG) were similar on LB and PB for strains with detectable activities (Figures 2F,G), the averages were significantly lower on PB when including strains with zero activity (*p* = 0.001 and *p* = 0.045, respectively, Wilcoxon test).

In LB, protein levels ranged from non-detectable for 13 strains to 1,177 μg protein mg^−1^ biomass for Betaproteobacteria strain 132 (Figure 2J). Protein levels could not be measured accurately in PB due to interference from the medium.

### Relationships Between Traits

**Figure 3:**
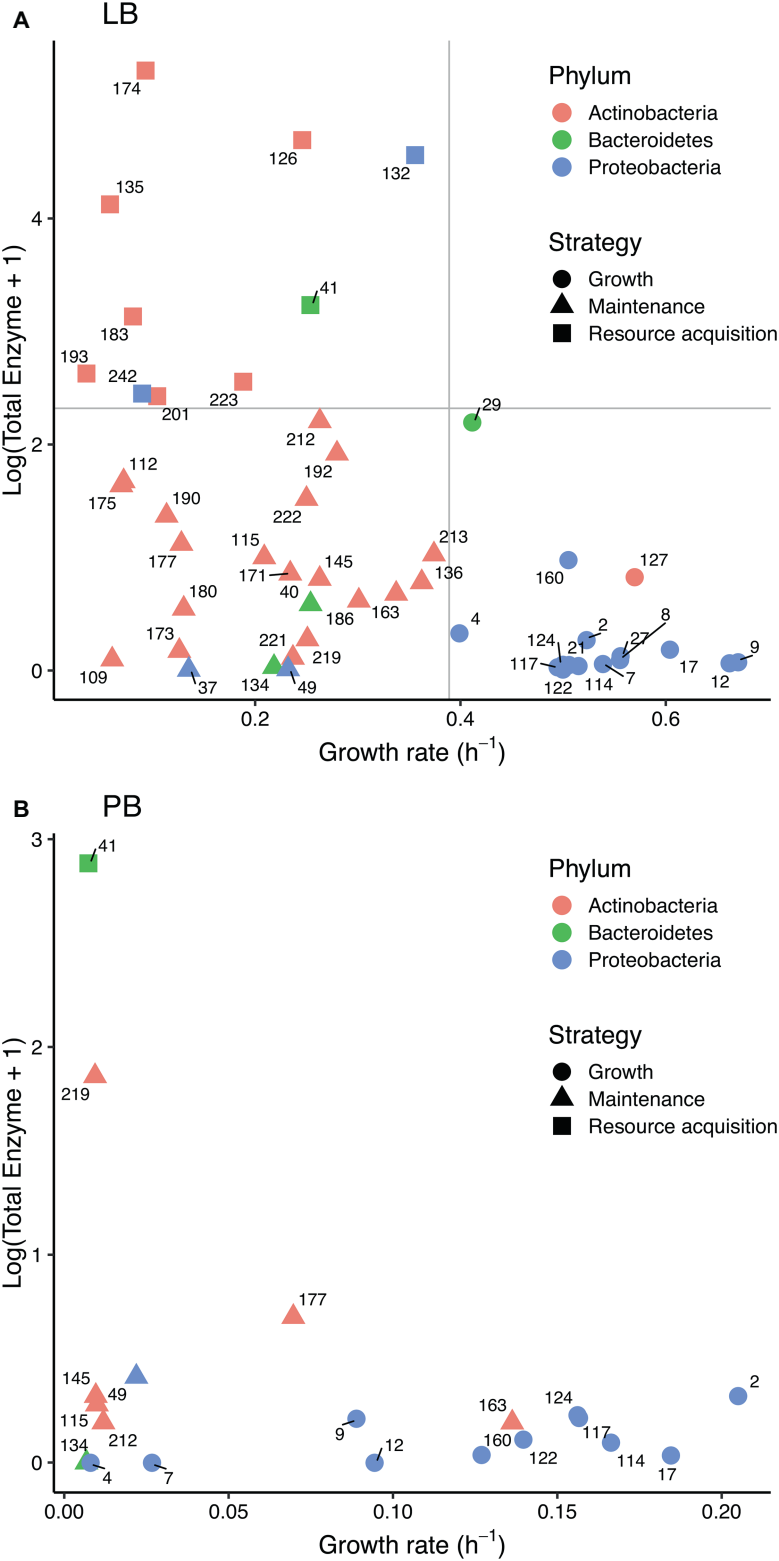
Relationship between (log+1)-transformed total extracellular enzyme activity and bacterial growth rate in **(A)** Luria broth and **(B)** plant litter broth. Colors correspond to bacterial phyla, and shapes correspond to life history strategies in Luria broth. Strain numbers are shown next to symbols, and gray lines in **(A)** denote cutoffs between strategies.

Consistent with a tradeoff, we found a negative correlation between total enzyme activity and growth rate in LB (Spearman *ρ* = −0.48, *p* < 0.001; Figure 3A); there was no significant relationship in PB (Figure 3B). There was also no significant relationship between protein level and growth rate in LB.

Based on Blomberg’s and Pagel’s tests of phylogenetic signal (Freckleton et al., 2002; Blomberg et al., 2003), the tradeoff between enzyme activity and growth rate may be driven by phylogenetic differences. Both tests indicated significant phylogenetic signal in growth rate and total enzyme activity on LB and PB (Table 1). Furthermore, the negative correlation between total enzyme activity and growth rate weakened when we accounted for phylogenetic signal (Pearson’s *r* = −0.22). Multiple individual enzymes showed significant phylogenetic signal based on Pagel’s *λ* in both media types (Table 1), but Blomberg’s *K* was only significant for LAP on LB and the sum of carbon-degrading enzymes on PB. In addition, Pagel’s *λ* indicated significant phylogenetic signal in extracellular protein levels on LB.

**Table 1:** Blomberg’s and Pagel’s phylogenetic signal of traits across 49 strains of bacteria growing on Luria broth (LB) or 20 strains growing on plant litter broth (PB).

	LB				PB			
	**Blomberg’s *K***	***p***	**Pagel’s *λ***	***p***	**Blomberg’s *K***	***p***	**Pagel’s *λ***	***p***
Growth rate	0.00750	**0.001**	1.093	**0.001**	0.0123	**0.009**	1.120	**0.001**
All enzymes	0.00367	**0.020**	0.895	**0.001**	0.0423	**0.028**	0.715	**0.001**
AG	0.00325	0.059	0.228	**0.001**	0.6378	0.061	1.004	**0.001**
AP	0.00834	0.125	0.037	0.365	0.0038	0.151	0.235	0.187
BG	0.00073	0.335	0.907	**0.001**	0.0261	0.245	0.121	0.405
BX	0.00038	0.614	0.113	0.471	0.3550	0.197	5.1 × 10^−5^	1.000
CBH	0.01791	0.096	1.110	**0.001**				
LAP	0.00471	**0.030**	0.392	**0.001**	0.0180	0.262	0.445	**0.048**
NAG	0.00107	0.302	0.082	0.057	0.0045	0.654	5.5 × 10^−5^	1.000
C enzymes	0.00232	0.075	0.830	**0.001**	0.4018	**0.013**	1.040	**0.001**
Protein	0.00101	0.137	0.853	**0.001**				

*p < 0.05, shown in bold*.

*On PB, CBH could not be analyzed due to low enzyme activities, and protein was not measurable*.

Based on the distribution of total enzyme and growth traits on LB, we assigned strains to growth, maintenance, and enzyme production strategies (Figure 3). Strategy was significantly correlated with phylum (*χ*^2^ = 27.521, df = 4, *p* < 0.001) and class (*χ*^2^ = 47.104, df = 12, *p* < 0.001). Of 16 growth strategists, 14 were Gammaproteobacteria such as *Pseudomonas* and *Erwinia*. Of the 23 maintenance strategists and 10 resource acquisition strategists, 26 were Actinobacteria. Eleven of the 20 strains that grew on PB were growth strategists, and all of those were Gammaproteobacteria. Only one resource acquisition strategist—a *Flavobacterium*—grew on PB.

## Discussion

Relatively few other studies have measured tradeoffs among maximum growth rate and enzyme production (Malik et al., 2019a). In support of our first hypothesis, we found a negative correlation between these two traits for strains growing on LB. However, there was no significant negative relationship between these variables on PB, the more resource-poor medium. This finding contrasts with our second hypothesis that predicted a stronger tradeoff under low resource conditions. Still, growth and enzymatic traits on both media types were phylogenetically structured, consistent with our third hypothesis. These results imply that bacteria have different life history strategies that are evolutionarily conserved and reflect a tradeoff between growth and enzyme production.

### Trait Tradeoffs Across Media Types

No strains on LB or PB displayed rapid growth along with high enzyme activity (Figure 3), likely due to the physiological costs of enzyme production. Such tradeoffs among traits can occur due to allocation of finite resources to different metabolic pathways (Treseder et al., 2011). Extracellular enzyme production requires metabolic investment in transcription, protein synthesis, and secretion (Glenn, 1976). Respiratory and anabolic costs associated with this investment could limit the resources available for biomass growth that requires cellular investment in biosynthesis, ribosomes, and DNA replication (Elser et al., 2000). These costs were probably associated with constitutive enzyme production because LB is an available substrate that can be metabolized without extracellular enzymes.

We analyzed bacterial traits on PB to quantify tradeoffs under resource poor conditions more typical of the litter environment from which our strains were isolated. Yet under these conditions, we did not detect the hypothesized tradeoff between growth and enzyme activity. One potential reason is that PB, while chemically different from LB, is not representative of field resource conditions. Plant litter in the field is composed of mainly insoluble polymers that require extracellular enzyme degradation (Alster et al., 2013), whereas PB contains mainly soluble monomers. An abundance of monomers relative to polymers in both media types might have suppressed inducible enzyme production and weakened tradeoffs that normally operate under field conditions. Indeed, many strains did not produce detectable enzyme activities on LB or PB, and most strains that grew on PB were growth strategists rather than resource acquisition strategists. Of these growth strategists, many were pseudomonads (Proteobacteria) that are known to thrive on the amino acid substrates present in LB and likely also PB (Jacoby, 1964).

### Life History Strategies

Our three proposed life history strategies, driven by trait data, share some characteristics with previous life history concepts for microbes, such as the copiotroph-oligotroph spectrum. Our growth strategy is similar to copiotrophy primarily because it is characterized by a high growth rate. Additionally, although copiotrophs are poor competitors in low resource environments, they can maintain some growth because they are classified as resource generalists with higher catabolic diversity (Freilich et al., 2009). Eleven of the 16 growth strategists were capable of growth in PB, a higher fraction than any of the other strategies, lending support to this idea.

Our maintenance strategists resemble oligotrophs owing to their slow growth rate, low extracellular enzyme activity, and possible resource specialization (Freilich et al., 2009). On the other hand, these traits may just reflect low performance on LB media. Still, some maintenance strategists could be classified as oligotrophs if they grow under low-resource conditions, such as strains 163 (*Curtobacterium*) and 177 (*Arenivirga*) that grew relatively well on PB. Other maintenance strategists might function as copiotrophs in resource-rich environments other than LB.

Maintenance strategists might invest in traits aside from extracellular enzymes or growth machinery. For example, some bacteria invest in cell walls, extracellular polymeric substances, or stress tolerance proteins to survive environments with high temperature and low moisture (Schimel, 2018), conditions that are common in the environment from which our strains were isolated. Alternatively, maintenance strategists might have been upregulating transport machinery or investing in membrane-tethered enzymes (Traving et al., 2015). Enzymes that are attached to the membrane would not be detected in our assays of culture supernatant. The maintenance strategy might also include bacteria with high growth yield but low growth rates, consistent with rate-yield tradeoffs (Lipson, 2015).

It is more difficult to place our resource acquisition strategists along the copiotroph-oligotroph spectrum. They have low growth rates like oligotrophs, but they also have high metabolic output in the form of extracellular enzymes. If we define resource availability in terms of monomers, then resource acquisition strategists are potential oligotrophs because extracellular enzymes allow for growth under low monomer conditions.

Parts of Grime’s competitor-stress tolerator-ruderal framework might also be relevant to the bacteria in our study. Our growth strategy is potentially analogous to Grime’s ruderal strategy. For microbes, ruderal traits can include increased investment in ribosomes, nucleotides, and central metabolic fluxes that are generally also required for high maximum growth rates (Wood et al., 2018). It is unclear whether our resource acquisition strategists align with Grime’s competitors, which out-compete other organisms for local resources. Investment in extracellular enzymes does not necessarily imply superior competitive ability. Cheaters—microbes that take up reaction products without producing their own enzymes—may be better competitors than enzyme producers, particularly under relatively well-mixed, high-resource conditions (Allison, 2005).

Challenges with applying CSR to microbes have led to new variations on Grime’s triangle. The YAS framework includes Grime’s stress tolerator strategy but defines two additional strategies based on resource allocation traits (Malik et al., 2020). The high-yield (Y) strategy is based on traits that maximize cellular biosynthesis relative to respiration and other biomass loss pathways. The resource acquisition (A) strategy is defined by traits like investment in extracellular enzymes and uptake transporters that facilitate resource acquisition.

Although our resource acquisition strategy fits with YAS, our growth and maintenance strategies do not line up as well. Growth rate is not a defining trait in YAS but rather an emergent property of other traits, and we did not measure yield. Neither did we measure stress tolerance directly, so it remains unclear if our maintenance strategists showed low growth and low enzyme production due to investment in stress tolerance traits.

In order to test YAS or Grime’s CSR triangle comprehensively, additional studies should measure microbial traits related to yield and stress tolerance under a wider range of conditions. For example, a future experiment could manipulate water potential of the growth environment and measure osmolyte or extracellular polymeric substance production. Ideally, this experiment could be done on more realistic growth substrates and include simultaneous measurements of growth yield. Additional experiments could test if tradeoffs are expressed over time, for example, with periods of microbial growth preceding or following the periods of enzyme production.

### Phylogenetic Conservation of Traits and Strategies

We found evidence that life history strategies and their underlying traits were phylogenetically conserved. Most Proteobacteria were growth strategists, whereas most Actinobacteria and Bacteroidetes were maintenance and resource acquisition strategists. Overall, the physiological differences between Proteobacteria and Actinobacteria contributed substantially to the growth-enzyme tradeoff that we observed on LB (Figure 3A). Some previous studies also suggest that life history strategies are conserved at the phylum level, specifically those along the copiotroph-oligotroph spectrum (Fierer et al., 2007; Philippot et al., 2010).

There were also notable patterns in bacterial traits below the phylum level. The Proteobacteria growth strategists were mainly pseudomonads, common inhabitants of the phyllosphere and fresh leaf litter that contains monomeric resources to support growth (Mouginot et al., 2014; Tláskal et al., 2016). We analyzed seven Actinobacteria from the genus *Curtobacterium*, all of which were classified as maintenance strategists. This genus is also common in the phyllosphere and surface litter (Chase et al., 2016), particularly in our field site where it can be a dominant bacterial lineage (Matulich et al., 2015). Previous analyses indicate that these *Curtobacteria* are tolerant of seasonal drought and have the capacity to degrade polymeric carbohydrates (Chase et al., 2017), although their C-degrading enzyme production was inconsistent in our study (Supplementary Material).

The strains we analyzed covered a wide range of enzymatic trait space. Strain 41, a member of the Bacteroidetes and a resource acquisition strategist, stood out for its high investment in extracellular enzyme activity and protein in both media types. A sister species within the same genus possesses many genes for peptidases and polysaccharide-degrading enzymes, including hemicellulases and chitinase, and produces high amounts of protein for use in motility (McBride et al., 2009). Although we observed a wide range of enzyme trait values, the phylogenetic diversity among our strains was limited, and a greater diversity of strains should be analyzed to verify our conclusions.

### Implications

The tradeoffs and life history strategies that we identified will likely have implications for ecosystem processes such as decomposition and carbon cycling (Krause et al., 2014). Bacterial communities dominated by resource acquisition strategies, and environmental conditions that select for them, should have relatively high rates of polymer decomposition and nutrient cycling due to the action of extracellular enzymes (Carreiro et al., 2000). Dominance by growth strategists could lead to fast processing of monomeric organic substrates and formation of more stable microbial biomass residues (Kallenbach et al., 2019). Carbon and nutrient processing by maintenance strategists might be more limited but relevant if these bacteria have mechanisms to tolerate stress and maintain biogeochemical functioning under harsh environmental conditions (Schimel et al., 2007). At the same time, microbes with different strategies can co-exist and even facilitate one another while driving processes like decomposition (Folse and Allison, 2012; Allison and Goulden, 2017). Given the importance of these ecosystem consequences, studies with more microbial strains, environmental factors, and trait measurements are warranted to test emerging conceptual models of microbial life history strategies.

## Data Availability Statement

All datasets generated for this study are included in the article/Supplementary Material.

## Author Contributions

KR contributed to project design, data collection, data analysis, and manuscript writing. SA contributed to project design, data analysis, and manuscript writing.

### Conflict of Interest

The authors declare that the research was conducted in the absence of any commercial or financial relationships that could be construed as a potential conflict of interest.

## Footnotes

^1^https://www.ebi.ac.uk/Tools/msa/mafft/

^2^https://itol.embl.de/
