# Supplementary material for: Bacterial Tradeoffs in Growth Rate and Extracellular Enzymes
Source: Front Microbiol. 2019 Dec 20;10:2956. doi: 10.3389/fmicb.2019.02956 (PMC6933949; doi:10.3389/fmicb.2019.02956)
Supplement: Supplementary file 1 [file Image_1.pdf]

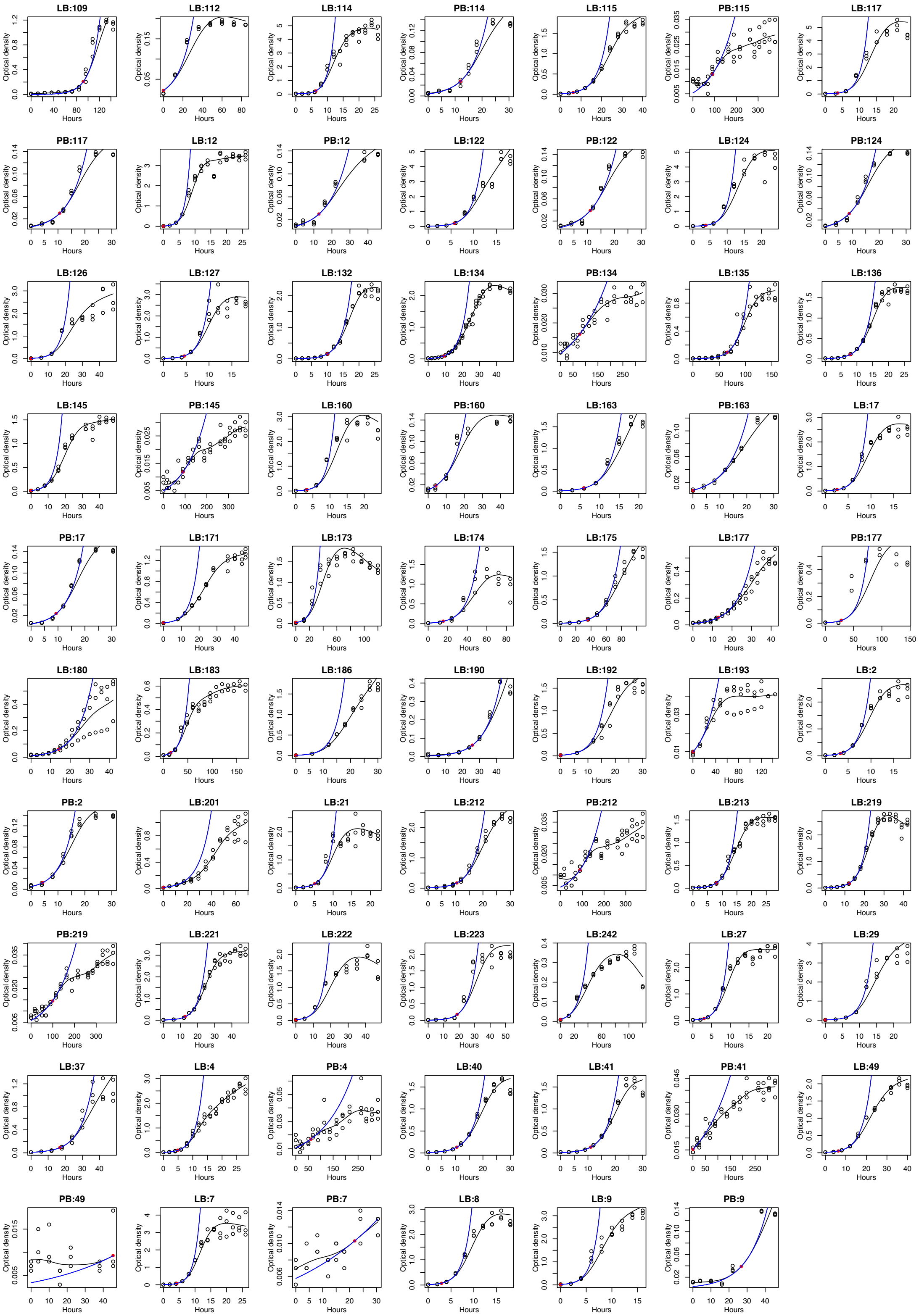

Supplementary Figure 1. Growth curves for bacterial strains on Luria broth (LB) or plant litter broth (PB). Strain numbers are shown over each panel. Black curves show the spline fit from the 'growthrates' R package; blue curves are the exponential functions used to derive growth rates.
